# Quitline Information Included on Cigarette Packaging: An Assessment of Country Adherence to WHO FCTC Guidelines, 2007 to 2018

**DOI:** 10.3390/ijerph182212193

**Published:** 2021-11-20

**Authors:** Christopher M. Seitz, Kenneth D. Ward, Zubair Kabir

**Affiliations:** 1 Department of Health & Exercise Science, Appalachian State University, Boone, NC 28608, USA; 2 Division of Social and Behavioral Sciences, The University of Memphis School of Public Health, Memphis, TN 38152, USA; kdward@memphis.edu; 3 School of Public Health, University College Cork, T12 XF62 Cork, Ireland; z.kabir@ucc.ie

**Keywords:** cigarettes, FCTC, quitline, warning

## Abstract

The purpose of this study is to evaluate country adherence to the World Health Organization’s (WHO) Framework Convention on Tobacco Control (FCTC) guidelines in terms of including quitline information on cigarette packaging. Data were gathered from the WHO’s Global Health Observatory database. The study included countries that were signatories to the FCTC, had a toll-free quitline, and required health warnings on cigarette packaging. Countries were then classified by income level according to the World Bank. From 2007 to 2018, the number of countries that established a quitline increased from 34 to 60. During the same timeframe among those countries, the number of countries that included information about the quitline on cigarette packaging increased from 5 to 37, with a larger proportion (79%) of high-income countries promoting their quitlines on cigarette packaging compared to middle-income (45%) countries. Although there was an increase in adherence to the WHO FCTC guidelines, there is still a need for several countries to include quitline information on cigarette packaging.

## 1. Introduction

In 2003, the World Health Organization (WHO) responded to the global consequences of tobacco use by creating the Framework Convention on Tobacco Control (FCTC). As an international public health treaty, the FCTC requires signatories to implement a variety of policies that have been shown to be effective at reducing both the supply and demand of tobacco products [1]. As of 2021, 182 countries have signed as parties to the treaty [2].

In 2008, the WHO developed “MPOWER,” which was a way to streamline the FCTC for practical implementation. Essentially, the acronym condenses the FCTC into six evidence-based strategies: Monitor tobacco use, Protect people from tobacco smoke, Offer help to quit tobacco use, Warn about the dangers of tobacco, Enforce bans on tobacco advertising and promotion, and Raise taxes on tobacco products [3]. The WHO determines each country’s level of MPOWER implementation [4] and several research studies have shown that there is a strong relationship between countries with high implementation of MPOWER and low smoking rates [5,6,7,8,9,10,11].

According to Article 14 of the FCTC, along with the “O” in MPOWER, it is recommended that countries create toll-free quitlines [12]. Quitlines are telephone-based counseling services in which trained cessation specialists provide advice on how to quit smoking. The use of quitlines increases quit rates by four percent, which is twice the rate compared to quitting without help [13]. Article 14 does not include a timeline for implementing its recommendations.

According to Article 11 of the FCTC, along with the “W” in MPOWER, it is recommended that countries who have quitlines advertise their quitlines along with graphic warnings on cigarette packaging [14]. Research shows that when promoting a quitline on cigarette packaging in combination with graphic pictorial health warnings, there may be a synergistic effect that can increase knowledge about quitlines [15,16,17,18,19], intention to quit [20], perceptions of health warning effectiveness [21], and calls to quitlines [22,23,24,25,26,27]. Article 11 states that cigarette packaging and labelling measures should be implemented within three years after ratifying the FCTC [14].

Although the WHO FCTC recommends placing quitline information on cigarette packaging, there is yet to be a formal assessment of how many countries are doing so. Therefore, the purpose of this study was to evaluate which of the nations that have ratified the FCTC have quitlines and place quitline information on cigarette packaging. In addition, this study sought to determine whether national quitline information is paired with graphic pictorial health warnings or if the quitline is listed along with a simple text-based health warning.

## 2. Materials and Methods

The sample for this study was determined using the WHO’s Global Health Observatory (GHO) database [28]. Countries were included in the study if they were signatories to the FCTC, had a toll-free quitline [29], and required health warnings on cigarette packaging [30]. The GHO was then used to determine how many countries in the sample included a quitline telephone number on cigarette packaging [31] and whether a graphic pictorial warning was also placed on the packaging [32].

Countries were then classified using the World Bank’s categories of income level. The World Bank divided country income groups by the 2020 gross national income per capita of: $1045 or less (low income), $1046 to $4095 (lower middle income), $4096 to $12,695 (upper middle income), and $12,696 or more (high income) [33]. For this study, a single “middle” income level was created by combining the lower middle and upper middle countries.

Finally, countries that had a quitline but did not include quitline information on cigarette packaging were then assessed regarding the year that the quitline was established and the year that the WHO FCTC was ratified. The GHO was used to determine the years that quitlines had been established [29]. The WHO’s 2019 “Report on the Global Tobacco Epidemic” was then used to determine dates that the FCTC was ratified by countries, which is the formal act of a country indicating their consent to be obligated by the treaty [14].

## 3. Results

From 2007 to 2018, the number of countries that established a quitline increased from 34 to 60. During the same timeframe among those countries, the number of countries that included information about the quitline on cigarette packaging increased from 5 to 37 (Table 1). As of 2018, a larger proportion (79%) of high-income countries were promoting their quitlines on cigarette packaging compared to middle-income countries (45%) (Table 1). In addition, by 2018, all countries that advertised their quitlines on cigarette packaging paired the information with graphic pictorial health warnings (Table 1). From 2007 to 2018, there were no low-income countries with an established quitline (Table 1).

Of the countries that did not advertise their quitline on cigarette packages, the difference in time between ratifying the FCTC and establishing a quitline ranged from 2 to 13 years with a mean of 7.2 years. There were six countries that established a quitline as early as 2007 and five countries that established a quitline as recently as 2018 (Table 2).

## 4. Discussion

This paper describes the first evaluation of country adherence to the WHO FCTC’s recommendation that quitline information be included on cigarette packaging. The findings indicate that since 2007, there has been an increase in the number of countries that establishing quitlines and promoting quitline use on cigarette packaging alongside graphic pictorial health warnings. However, this study found that 23 countries with an established quitline did not include quitline information on cigarette packaging, even though those countries ratified the FCTC an average of 15 years ago. This study also found that a larger proportion of countries with high income levels used cigarette packaging to promote their quitlines, which reflects previous findings that country development is associated with level of MPOWER implementation [5,6,7], including the establishment of quitlines [34] and health warnings on cigarette packaging [35].

The findings highlight the need for signatories to implement the measures specified in Articles 11 and 14 of the FCTC. As previous research has found, there are several parties to the FCTC that have yet to establish national toll-free quitlines [34]. Of the 182 parties to the treaty, only 60 (32%) in this study have established quitlines since 2007. Among those, only 37 (61%) include quitline information on cigarette packaging. When ratifying the FCTC, parties agree to adopt and implement the measures specified in Article 11 regarding cigarette packaging and labelling within a three-year period [13]. Several parties in this study ratified the FCTC and established their quitlines several years ago, meaning that the parties have not adhered to the timeline specified in the treaty. This reflects the lack of full implementation of FCTC among several nations to date.

The findings from this study have practical implications for tobacco control. The leaders of tobacco control from each party should advocate that quitine information be added to the mandated health warnings on cigarette packaging. By simply adding the quitline telephone number to existing health warnings on cigarette packaging, research suggests that calls to the quitline will increase [22,23,24,25,26,27]. Adding quitline information to health warnings is a zero-cost approach to help facilitate the use of the quitline by those who desire to quit smoking [25]. Research suggests that the quitline number should be located on the front and/or back of cigarette packages [17,20,25], should be large enough to be visually salient [20], and may include a positive message [19,21,23] such as “You can quit smoking. Call quitline” [20] or “Want advice on quitting? Call quitline” [17].

This study’s findings also reflect a broader discussion regarding the barriers that low-income and middle-income countries may experience when establishing quitlines and advertising those quitlines on cigarette packaging. With regard to adopting quitlines, the largest disparity is found within low-income and middle-income countries [13], which may be due to a lack of available government funds to create and maintain a national quitline [36]. Fortunately, text messaging and app-based smoking cessation interventions have been developed with the growth of mobile phone technology. Research indicates that these interventions could be an option for low-income and middle-income countries as the interventions are not only effective but also cost less and require fewer resources than traditional quitlines [13,37,38]. In addition, the WHO and other tobacco control experts have also recommended partnerships with the private sector, excluding the tobacco industry, to help fund quitlines and include various stakeholders that would benefit from their employees being healthy and smoke-free [13,36,39].

In terms of advertising established quitlines along with health warnings on cigarette packaging, it is possible that countries may experience pushback from the tobacco industry. Research strongly indicates that the tobacco industry is aggressive in advocating that policymakers not include, or not enhance, health warnings on cigarette packaging [36,40,41,42,43]. The industry tends to make false claims (e.g., warnings are not necessary, too expensive, or take a long time to implement) in order avoid policies that mandate health warnings on cigarette packaging [40]. In response, public health professionals should counter the industries’ methods by also advocating to policymakers. Advocacy efforts should include correct information about the overwhelming evidence in support of the need for, and the effectiveness of, quitline information and health warnings placed on cigarette packaging [36,40,43]. Tobacco control advocates should consider partnering with the WHO and/or the organization ”STOP”, which specializes in working with low-income and middle-income countries to work against tobacco industry efforts [44].

The limitations of this study should be taken into consideration when interpreting the findings. First, the GHO reports data that were collected every other year. The repository also does not specify the year that a quitline was established but simply lists which countries do or do not have a quitline during the year of data collection. As such, it is possible that the years listed in this study regarding quitline establishment may be inaccurate and that quitlines were established the year prior to the year listed. In addition, it is possible that parties established quitlines prior to 2007. Second, the latest data posted on the GHO regarding the variables in this study were from 2018. It is possible that more countries have developed quitlines and included information about the quitlines on cigarette packaging since 2018.

## 5. Conclusions

There has been an increase in adherence to the WHO FCTC guidelines regarding the establishment of quitlines and promoting these quitlines on cigarette packaging. However, there is still a need for countries to place information about their quitlines on cigarette packaging. Leaders in tobacco control from each country should advocate the inclusion of quitline information on cigarette packaging as it is considered an evidence-based, zero-cost approach to promoting smoking cessation.

## Figures and Tables

**Table 1:** Countries that have quitlines and cigarette packaging that includes quitline information with or without a graphic pictorial by income level, 2007–2018.

Country Income Level, Quitline Information	2007	2008	2010	2012	2014	2016	2018
High income Established toll-free quitline Packaging includes quitline with pictorial Packaging includes quitline with no pictorial	25 4 1	26 4 1	27 5 1	27 7 2	25 7 2	28 19 1	29 23 0
Middle income Established toll-free quitline Packaging includes quitline with pictorial Packaging includes quitline with no pictorial	9 1 0	13 3 1	16 3 1	19 7 1	20 7 1	27 9 1	31 14 0
Low income Established toll-free quitline Packaging includes quitline with pictorial Packaging includes quitline with no pictorial	0 NA NA	0 NA NA	0 NA NA	0 NA NA	0 NA NA	0 NA NA	0 NA NA
Total Established toll-free quitline Packaging includes quitline with pictorial Packaging includes quitline with no pictorial	34 5 1	39 7 2	43 8 2	46 14 3	45 14 3	55 28 2	60 37 0

Abbreviation: NA, not applicable.

**Table 2:** Countries with quitlines that do not include quitline information on cigarette packaging, by income level, year of FCTC ratification, and year the quitline was established.

Income Level	Country	FCTC Ratification	Quitline Established
High	Czechia Estonia Finland Kuwait Saudi Arabia United Arab Emirates	2012 2005 2005 2006 2005 2005	2018 2007 2007 2012 2018 2007
Medium	Azerbaijan Belarus Bhutan Brazil Cote d’Ivoire Cameroon Honduras Iran Jamaica Kenya Peru Russian Federation Sri Lanka Tonga Turkmenistan Ukraine Vietnam	2005 2005 2004 2005 2010 2006 2005 2005 2005 2004 2004 2008 2003 2005 2011 2006 2004	2016 2018 2014 2007 2016 2016 2007 2007 2014 2014 2014 2010 2010 2016 2018 2018 2016

## Data Availability

The data used for this study are publicly available and listed in the citations within the “Materials and Methods” section of the paper.
